# Synthesis and Characterization of Novel Hydrazone Derivatives of Isonicotinic Hydrazide and Their Evaluation for Antibacterial and Cytotoxic Potential

**DOI:** 10.3390/molecules27196770

**Published:** 2022-10-10

**Authors:** Muhammad Abdullah Shah, Ala Uddin, Muhammad Raza Shah, Imdad Ali, Riaz Ullah, Peer Abdul Hannan, Hidayat Hussain

**Affiliations:** 1 Department of Chemistry, Bacha Khan University, Charsadda 24420, Pakistan; 2 Department of Chemistry, University of Buner, Buner 19290, Pakistan; 3 H.E.J. Research Institute of Chemistry, International Center for Chemical and Biological Sciences, University of Karachi, Karachi 74200, Pakistan; 4 Department of Pharmacognosy, College of Pharmacy, King Saud University, Riyadh 11421, Saudi Arabia; 5 Department of Pharmacy, Sarhad University of Science and Information Technology, Peshawar 25000, Pakistan; 6 Department of Bioorganic Chemistry, Leibniz Institute of Plant Biochemistry, Weinberg 3, D-06120 Halle (Salle), Germany

**Keywords:** azomethine, cytotoxicity, hydrazones, minimum inhibitory concentration, pharmacological potential

## Abstract

Hydrazones are active compounds having an azomethine –NHN=CH group and are widely studied owing to their ease of preparation and diverse pharmacological benefits. Novel isonicotinic hydrazone derivatives of vanillin aldehyde and salicyl aldehyde were synthesized that had azomethine linkages and were characterized by UV–Visible, FTIR, EI-MS, 1H-NMR and 13C-NMR spectroscopy. The compounds were screened for their antibacterial activity against Staphylococcus aureus, Bacillus subtilus, and Escherichia coli using disc diffusion and minimum inhibitory concentration (MIC) methods. For cytotoxicity, a brine shrimp lethality test was performed to calculate the lethal concentration (LC50). The results demonstrated appreciable antibacterial activities against the applied strains, amongst which the compounds coded NH3 and NH5 showed maximum inhibition and MIC responses. In terms of cytotoxic activity, the maximum effect was observed in compound NH5 and NH6 treatments with minimum survival percentages of 36.10 ± 3.45 and 32.44 ± 2.0, respectively. These hydrazones could be potential candidates in antitumorigenic therapy against various human cancer cells.

## 1. Introduction 

New complex diseases are likely to affect humans because of changing global climatic conditions [1]. There is a continuous need to discover new remedies to combat diseases caused by resistant pathological strains to improve human life [2]. A variety of compounds have been synthesized or isolated from various natural sources, the majority of which have been reported to have significant therapeutic potentials [3,4,5,6,7]. Amongst them, the hydrazones, a class of synthetic organic compounds, are being recognized due to their wide range of pharmacological benefits, including antimicrobial, anti-inflammatory, antifungal [8], anticancer, antioxidant, cardioprotective, antiprotozoal and many other activities [9,10,11]. These compounds possess an –HC=N–NH– bond, which is the active site [12], and further combining hydrazones with numerous functional groups leads to the formation of products with unique biological properties [13]. 

Hydrazones possesses some particular properties which make them a potential candidate for designing new moieties. They contain a C=N bond in conjugated form with a functional nitrogen electron pair. They are distinguished from other members of this class (imines, oximes) by the presence of two interlinked nitrogen atoms [14,15,16]. These nitrogen atoms are nucleophilic, while the carbon has both an electrophilic and nucleophilic nature and further combining hydrazones with numerous functional groups leads to the formation of products with unique biological properties [13,17].

Isoniazid is a first-line antitubercular drug and it acts by inhibiting enoyl reductase (InHA) in *Mycobacterium Tuberculosis*. This drug is a prodrug and needs to be activated by KatG catalase-peroxidase [18]. A number of hydrazone derivatives have been synthesized from isoniazid and were found to have potentiated activities against various bacterial and fungal strains [19], including hydrazones with benzohydrazide and menthone [11,18,20]. Furthermore, metal complexes of isonicotinic hydrazones including, copper, zinc, manganese, nickel showed enhanced activities against microbes, tumor and free radicals [21,22]. 

**Figure 1:**
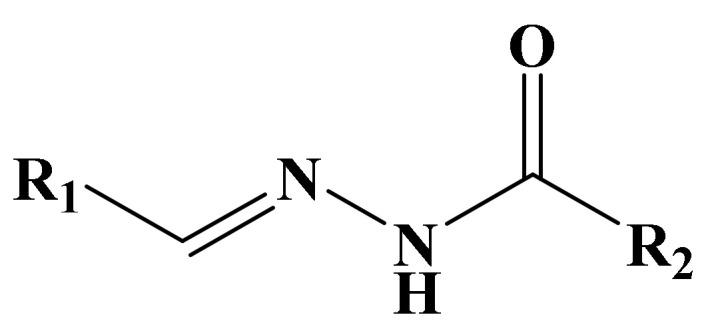
General Structure of Acylhydrazone. R_1_ is benzene and R_2_ is the pyridine ring.

Hydrazones are usually synthesized by heating hydrazides with different aldehydes using solvents such as acetone, ethanol that can be easily confirmed from its spectral data [9]. Further, they can be used as an intermediate in Wolff Kishner reductions to reduce the carbonyl functionality of aldehyde and ketones into alkanes [23]. Based on the aforementioned literature, the current research project has been designed to synthesize new hydrazone derivatives of alkylated vanillin aldehyde and salicyl aldehyde with isonicotinic hydrazide, with the aim to achieve biologically active derivatives having potential antibacterial and cytotoxic properties. The structure of acylhydrazone is given below in Figure 1.

## 2. Materials and Methods

### 2.1. Chemicals and Spectroscopic Analyses

Chemicals/reagents of analytical grade were purchased from Sigma Aldrich. These included vanillin, 4-hydroxybenzaldehyde, tertbutyl bromoacetate, 3,3-dimethylallyl bromide, 1-bromopropane, isoniazid, acetic acid, potassium carbonate, acetone, hexane, ethanol, ethyl acetate, chloroform, and double-distilled water.

Melting points were determined on an Electrothermal 9100 melting point apparatus (Weiss-Gallenkamp, Loughborough, UK). The purity of the compounds was confirmed by Thin-Layer Chromatography (TLC) using Kieselgel 60 F_254_ (Merck). EI-MS was performed on a JEOL JMS 600 mass spectrometer. ^1^H-NMR Bruker signals appeared at 300 and 400 MHz in CH_3_OH-*d_4_* and that of ^13^C-NMR at 75 MHz in CHCl_3_-*d_4_*. Fourier-transform infrared spectroscopy (FT-IR) spectra were obtained on a Shimadzu, IR Prestige- 21 (Shimadzu, Tokyo, Japan). 

### 2.2. General Procedure for the Synthesis of Isonicotinic Hydrazid-Based Hydrazone Derivatives 

**Figure 2:**
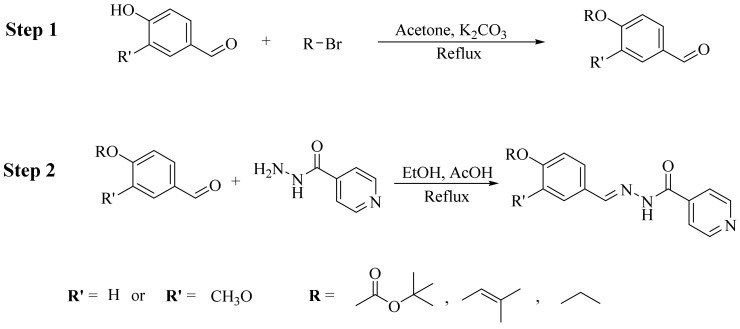
Synthesis of alkylated isoniazid derivatives.

The synthesis of isoniazid-based hydrazones was accomplished in 2 steps (Figure 2). In the first step, each 4-hydroxy-3-methoxy-benzaldehyde (Vanillin)/4-hydroxybenzaldehyde was alkylated with 5mmol of bromo alkyl derivatives and 5 mmol of K_2_CO_3_ in acetone as a solvent under reflux conditions. After completion of the reaction, the solvent was evaporated. The solid residue thus obtained was extracted three times with biphasic system of water and dichloromethane. The combined organic phase was evaporated over a rotary evaporator. The crude product was then subjected to column to obtain the desired product in a good yield. In the next step, 2 mmol of each alkoxy benzaldehyde derivative was reacted with 2 mmol of isoniazid in ethanol followed by the addition of a few drops of acetic acid. The reaction mixture was refluxed for 2.5 to 3 h and the progress of the reaction was observed via TLC. After the completion of the reaction, the precipitate was filtered, washed with cold distilled water and dried. The desired compound was further purified by washing it twice with 30% ethyl acetate: hexane. The products obtained were in excellent yields. The data are summarized in Table 1 and the compounds are shown as their code names (i.e., NH1-NH6).

#### 2.2.1. NH1 Tert-butyl (E)-2-(4-((2-Isonicotinoylhydrazineylidene) methyl)-2-methoxyphenoxy) Acetate

UV/Vis (DMSO): λ_max_ = 322 nm. IR (KBr, vmax/cm^−1^): 3230.42 (N-H), 1730.65 (C=O), 1639.49 (C=O), 1577.51 (C=N), 1163.08 (N-N). ^1^H-NMR (300 MHz, CH_3_OH-*d_4_*, δ/ppm): δ 8.745 (d, *J* = 3.6 Hz, 2H), 8.269 (s, 1H) 7.886 (d, *J* = 4.5 Hz, 2H), 7.747 (d, *J* = 1.2 Hz 1H), 7.187 (dd, *J* = 6.0, *J* = 1.2 Hz, 1H), 6.904 (d, *J* = 6.3 Hz, 1H), 4.654 (s, 2H), 3.934 (s, 3H), 1.475 (s, 9H). ^13^C-NMR chemical shift δ (ppm) (CHCl_3_-*d_1_*, 75.0 MHz): 168.70 (C-1), 164.14 (C-21), 152.72 (C-6), 150.41 (C-20), 150.17 (C-26), 143.27 (C-18), 141.21 (C-22), 126.84 (C-9), 123.42 (C-27), 121.75 (C-10), 116.13 (C-11), 115.07 (C-8), 82.17 (C-12), 68.38 (C-2), 57.62 (C-17), 28.55 (C-13,14,15). EIMS (m/z): 385.1 [M]^+^.

#### 2.2.2. NH2 Tert-butyl (E)-2-(4-((2-Isonicotinoylhydrazineylidene) methyl) phenoxy) Acetate

UV/Vis (DMSO): λ_max_ = 322 nm. IR (KBr, vmax/cm^−1^): 3294.42 (N-H), 2926.01 (aromatic C-H), 1733.15 (C=O), 1678.07 (C=O), 1612.49 (C=N), 1151.50 (N-N). ^1^H-NMR (300 MHz, CH_3_OH-*d_4_*, δ/ppm): δ 8.735 (dd, *J* = 4.5, 1.2 Hz, 2H), 8.297 (s, 1H), 7.874 (dd, *J* = 4.8, 1.5 Hz, 2H), 7.788 (dd, 6.2, 1.2 Hz 2H), 6.978 (dd, 6.6, 1.2 Hz, 2H), 4.645 (s, 2H), 1.484 (s, 9H). ^13^C-NMR chemical shift δ (ppm) (CHCl_3_-*d_1_*, 75.0 MHz): 168.70 (C-1), 164.14 (C-19), 162.89 (C-6), 150.41 (C-24), 143.97 (C-16), 141.21 (C-20), 130.83 (C-8), 125.87 (C-9), 123.42 (C-25), 114.72 (C-7), 82.14 (C-12), 68.38 (C-2), 28.25 (C-13, 14, 15). EIMS (m/z): 355.3 [M]^+^.

#### 2.2.3. NH3 (E)-N′-(3-Methoxy-4-((3-methylbut-2-en-1-yl)oxy)benzylidene)isonicotinohydrazide

UV/Vis (DMSO): λ_max_ = 330 nm. IR (KBr, vmax/cm^−1^): 3294.29 (N-H), 2935.86 (aromatic C-H), 1683.86 (C=O), 1590.27 (C=N), 1149.60 (N-N). ^1^H-NMR (300 MHz, CH_3_OH-*d_4_*, δ/ppm): δ 8.744 (d, *J* = 4.2 Hz, 2H), 8.265 (s, 1H), 7.885 (dd, *J* = 4.5, 1.2 Hz, 2H), 7.714 (d, 1.5 Hz 1H), 7.185 (dd, *J* = 7.5, 1.2 Hz, 1H), 6.982 (d, *J* = 6.0 2H), 5.506–5.466 (m, 1H), 4.612 (d, *J* = 5.1 Hz, 2H), 3.910 (s, 3H), 1.770 (d, *J* = 9.6 Hz, 6H). ^13^C-NMR chemical shift δ (ppm) (CHCl_3_-*d_1_*, 75.0 MHz): 164.08 (C-18), 152.17 (C-2), 150.40 (C-21), 148.73 (C-3), 143.19 (C-15), 141.13 (C-19), 132.73 (C-10), 128.21 (C-5), 123.98 (C-9), 123.43 (C-20), 121.98 (C-6), 117.19 (C-7), 114.91 (C-4), 67.21 (C-8), 56.75 (C-14), 23.93 (C-11), 17.21 (C-12). EIMS (m/z): 339.2 [M]^+^.

#### 2.2.4. NH4 (E)-N′-(4-((3-Methylbut-2-en-1-yl)oxy)benzylidene)isonicotinohydrazide

UV/Vis (DMSO): λ_max_ = 328 nm. IR (KBr, vmax/cm^−1^): 3237.52 (N-H), 2975.26 (aromatic C-H), 1745.10 (C=O), 1578.37 (C=N), 1112.43 (N-N). ^1^H-NMR (300 MHz, CH_3_OH-*d_4_*, δ/ppm): δ 7.877 (dd, *J* = 4.5, 0.9 Hz, 2H), 8.290 (s, 1H), 7.877 (dd, *J* = 4.5, 0.9 Hz, 2H), 7.766 (d, *J* = 6.6 Hz 2H), 6.976 (d, *J* = 6.6 Hz, 2H), 5.465–5.445 (m, 1H), 4.586 (d, *J* = 5.1 Hz, 2H), 1.780 (d, *J* = 8.1 Hz, 6H). ^13^C-NMR chemical shift δ (ppm) (CHCl_3_-*d_1_*, 75.0 MHz): 164.51 (C-16), 162.72 (C-2), 150.51 (C-19), 143.39 (C-13), 141.47 (C-17), 133.11 (C-10), 130.78 (C-6), 125.74 (C-5), 124.01 (C-9), 123.52 (C-18), 114.73 (C-7), 66.17 (C-8), 24.79 (C-11), 18.21 (C-12). EIMS (m/z): 309.2 [M]^+^.

#### 2.2.5. NH5 (E)-N′-(3-Methoxy-4-propoxybenzylidene) isonicotinohydrazide

UV/Vis (DMSO): λ_max_ = 328 nm. IR (KBr, vmax/cm^−1^): 3257.94 (N-H), 2942.74 (aromatic C-H), 1730.69 (C=O), 1613.26 (C=N), 1150.92 (N-N). ^1^H-NMR (400 MHz, CHCl_3_-*d_4_*, δ/ppm): δ 8.742 (dd, *J* = 4.5, 1.2 Hz, 2H), 8.262 (s, 1H), 7.884 (dd, *J* = 4.8, 1.2 Hz, 2H), 7.71 (d, *J* = 7.5 Hz, 1H), 7.185 (dd, *J* = 7.5, 1.2 Hz, 1H), 6.972 (d, *J* = 7.5, Hz, 1H), 4.0 (t, *J* = 5.1 2H), 3.913 (s, 3H), 1.856–1.804 (m, 2H), 1.051 (t, *J* = 5.7 Hz, 3H). ^13^C-NMR chemical shift δ (ppm) (CHCl_3_-*d_1_*, 75.0 MHz): 164.79 (C-16), 150.17 (C-3), 149.53 (C-2), 149.13 (C-21), 143.52 (C-13), 140.97 (C-17), 127.82 (C-5), 124.20 (C-22), 122.31 (C-6), 115.34 (C-7), 114.12 (C-4), 71.89 (C-8), 57.19 (C-12), 22.13 (C-9), 10.49 (C-10). EIMS (m/z): 313.2 [M]^+^.

#### 2.2.6. NH6 (E)-N′-(4-Propoxybenzylidene) Isonicotinohydrazide

UV/Vis (DMSO): λ_max_ = 320 nm. IR (KBr, vmax/cm^−1^): 3290.73 (N-H), 2975.35 (aromatic C-H), 1737.26 (C=O), 1588.12 (C=N), 1138.37 (N-N). ^1^H-NMR (400 MHz, CH_3_OH-*d_4_*, δ/ppm): δ 8.73 (d, *J* = 4.5 Hz, 2H), 8.29 (s, 1H), 7.87 (dd, *J* = 4.5, 0.9 Hz, 2H), 7.76 (d, *J* = 6.6 Hz 2H), 6.97 (d, *J* = 6.6 Hz, 2H), 3.96 (t, *J* = 5.1 Hz, 2H), 1.85–1.76 (m, 2H), 1.04 (t, *J* = 5.4 Hz, 3H). ^13^C-NMR chemical shift δ (ppm) (CHCl_3_-*d_1_*, 75.0 MHz): 168.08 (C-14), 161.71 (C-2), 151.13 (C-17), 144.02 (C-11), 142.25 (C-15), 129.66 (C-4), 125.10 (C-5), 122.79 (C-16), 115.21 (C-3), 72.63 (C-8), 22.84 (C-9), 10.91 (C-10). EIMS (m/z): 283.2 [M]^+^.

### 2.3. Antibacterial Activity

#### 2.3.1. Bacterial Strains Used

All the synthesized compounds were screened for antibacterial activities using minimum inhibitory concentrations (MIC) and zone of inhibitions against Gram (+)and Gram (−) bacterial strains. ATCC strains of the test microorganism selected for antibacterial assay were Gram-positive bacteria; *Staphylococcus aureus* ATCC 25923, *Bacillus subtilus* ATCC 11774, and Gram-negative bacteria; *Escherichia coli* ATCC 10536. The bacterial strains’ stock cultures were kept in Nutrient agar (Oxoid, UK) at 4 °C and inocula were prepared by transferring several single colonies of microbes to a sterile Mueller Hinton broth. The bacterial cell suspension was mixed until homogeneity to give a final density of 5 × 10^5^ cfu/mL

#### 2.3.2. Paper Disc Diffusion Method for Zone of Inhibition Assay

Preliminary screenings of synthesized compounds were conducted on agar plates using the disc diffusion method. The plates were prepared using Mueller–Hinton agar following the autoclave process (121 °C for 30 min). After cooling, the plates were inoculated with the suspension of microorganisms (*S. aureus, B. subtilis* and *E*. *coli*). The paper discs impregnated with the test compounds (1000 µg mL^−1^ in dimethyl sulfoxide) were placed on the solidified medium and incubated at 37 °C for 24 hours before recording the zone of inhibition in millimeters [24,25]. 

#### 2.3.3. Microplate Assay of Minimum Inhibitory Concentration (MIC)

The minimum inhibitory concentrations of test microorganisms and reference material were determined using the tetrazolium microplate assay method [26]. The assay was performed using a 96-well clear microtiter plate. Freshly harvested bacterial cell and fungal cell suspensions were seeded at 5 × 10^5^ cfu/mL in each well of the 96-well plate. Different concentrations, 500 to 10 µg/mL, of the test compounds were diluted in series with Muller–Hinton broth. A volume of 200 µL of each concentration was added in triplicate to the wells and the plates were then incubated for 18–24 h at 37 °C ± 0.5. After incubation, in each well, 50 µL of 3-(4, 5-dimethylthiazol-2-yl)-2, 5-diphenyltetrazolium bromide MTT, having a concentration of 0.2 mg/mL, was added and the plate was incubated at 37 °C for 30 min. An appropriate solvent blank (DMSO) was included as the negative control and the bacterial suspension was included as the positive control. The absorbance was measured at 570 nm with a reference wavelength of 650 nm by adding DMSO using a spectrophotometer and the percentage reduction of the dye (indicating the bacterial growth inhibition) was calculated [27].

#### 2.3.4. Morphological Changes Studied by Atomic Force Microscopy (AFM)

All the strains were grown on tryptic soya agar for 24 h at 37 °C. A volume of 10 µL of polylysine was added to the freshly cleaved mica slides and dried. A few drops of diluted culture, with a density of 10^5^ cfu, was applied to these slides and dried at an ambient temperature, and they were subjected to atomic force microscopy (AFM) for morphological analysis. For test compounds, 5–10 µL of samples was taken from MIC wells and applied on polylysine slides, dried and subjected to AFM. The morphological changes with and without test compounds were recorded and compared. 

### 2.4. Cytotoxicity

The cytotoxicities of synthesized hydrazones were recorded by employing an in vitro brine shrimp lethality bioassay technique [28]. The eggs of *Artemia salina* (brine shrimp) were allowed to incubate at 30 °C for 48 h under a light and dark cycle using artificial sea water before subjecting them to the sample solutions. The hatched larvae (nauplii) were collected with using a Pasteur pipette and were transferred to freshly prepared sea water (commercial salt and double-distilled water). The stock solutions of each compound were prepared at 500 µg/mL concentration in DMSO and sea water at 1:1, and further serial dilutions of 25, 50, 100, 200 and 300 µg/mL were prepared using sea water. Using a 2mL volume from each dilution, sea water containing 10 nauplii was added to a test tube and incubated for 24 h. A solvent mixture with nauplii containing no test compound was used as the control. After due time, the number of nauplii that survived was determined using a magnifying glass. The experiment was performed in triplicate and the median lethal concentration (LC_50_) ± SEM was calculated using regression analysis.

## 3. Results and Discussion

### 3.1. Antibacterial Assay

The antibacterial sensitivities of test compounds were assayed using the paper disc diffusion method using Amikacin for comparison, as shown in Table 2. From the screening, it was concluded that compounds show varied responses against S. *aureus*, *B. subtilis*, and *E. coli*. Against the *S. aureus* compounds NH1 and NH5, optimum inhibitions of 24 mm and 22 mm, respectively, were found. Against *B. subtilis*, the compound NH5 showed a maximum inhibition of 25 mm, while NH3, NH4 and NH6 presented 23 mm zones of inhibition. In the case of *E. coli,* the compound NH3 was found to have a significant inhibitory effect of 33 mm, while the compounds NH1 and NH5 showed 25 mm and 24 mm zones of inhibition, respectively. 

#### 3.1.1. Determination of Minimum Inhibitory Concentration (MIC)

**Figure 3:**
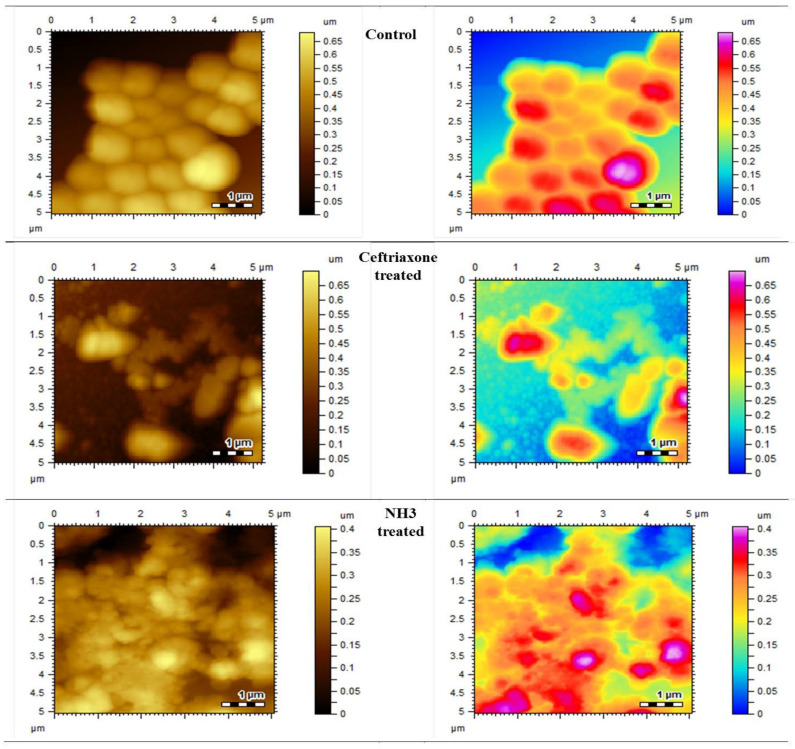
Atomic force microscopic images of control, standard and most effective treatment groups.

**Figure 4:**
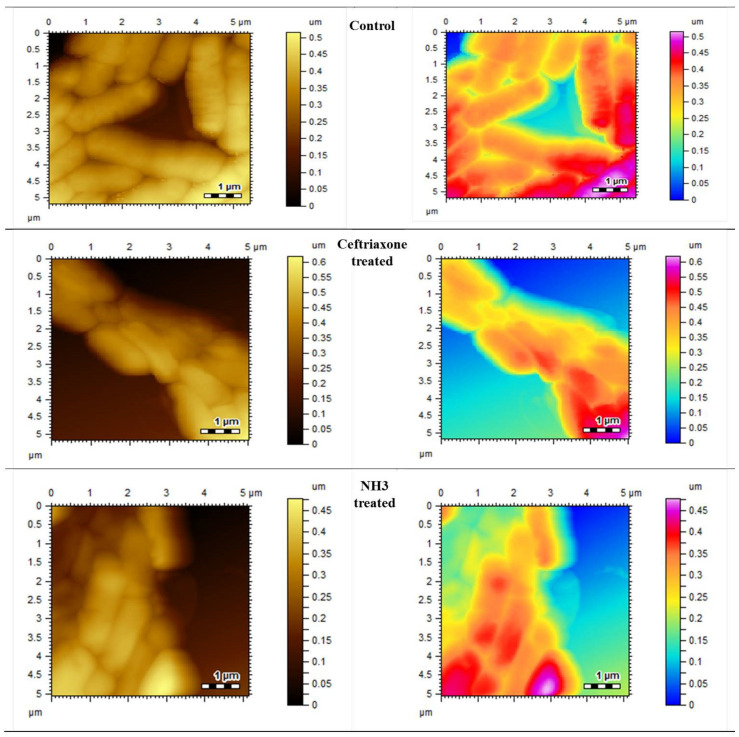
Atomic force microscopic images of control, standard and most effective treatment groups.

**Figure 5:**
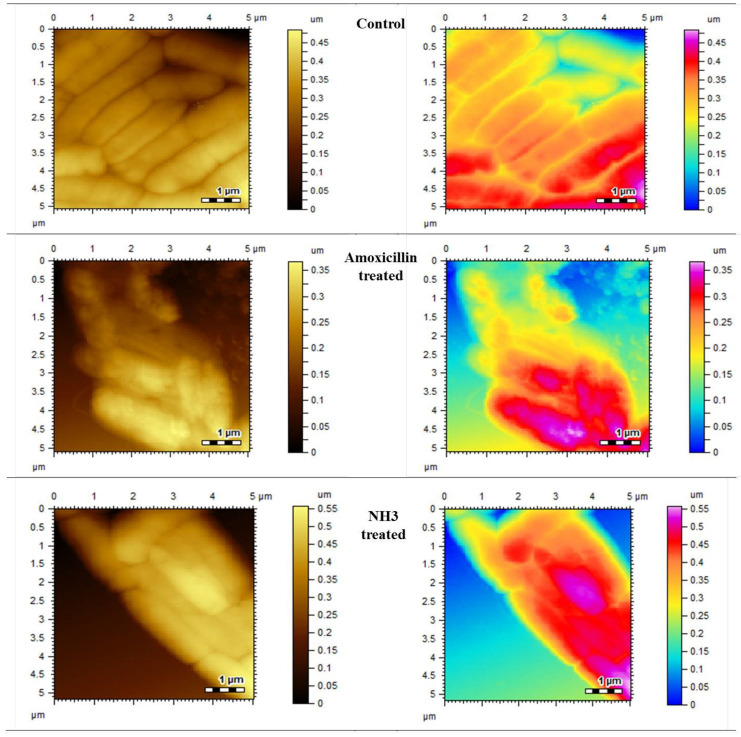
Atomic force microscopic images of control, standard and most effective treatment groups.

In order to determine the MIC of synthesized compounds, ceftriaxone and amoxicillin were used as standards to compare antibacterial effects. Ceftriaxone was used against *Staphylococcus aureus* and *Bacillus subtilis,* while amoxicillin was used against *Escherichia coli.* The compounds were tested in a concentration range of 25–500 µg/mL and the percent inhibitions were recorded as mentioned in Table 3. Ceftriaxone showed a significant effect with an IC_50_ of 50 µg/mL, inhibiting 55 ± 0.3% of *S*. *aureus,* and in the case of *B*. *subtilis,* the IC_50_ was 50 µg/mL inhibiting 55 ± 0.7% of the population (Figure 3 and Figure 4). Similarly, amoxicillin exhibited a significant effect against *E*. *coli* at IC_50_ of 50µg/mL inhibiting 66 ± 0.5% of microbial growth, as shown in Figure 5. 

The synthesized compounds also showed inhibition at various percentages. Among the synthesized compounds (NH1-NH6), the highest inhibitory effect was presented by NH3 against all bacterial strains. At a dose of 200 µg/mL, it inhibited the growth by 60 ± 1.5% of *S*. *aureus*, 53 ± 0.7% of *B*. *subtilis* and 65 ± 0.3 of *E*. *coli*, respectively, as mentioned in Table 3, Table 4 and Table 5. The variations in the cultures are visible and can be differentiated from the blank control by comparing the AFM images given in Figure 3 and Figure 4.

#### 3.1.2. Morphological Observations Using Atomic Force Microscopy

To visualize the surface morphological changes in bacterial cultures, the atomic force microscopy was utilized. AFM has the capability to record the changes in biological samples without any damaging effect. The AFM images of normal bacterial cultures of S. *aureus*, *B. subtilis* and *E. coli* were taken as mentioned in Figure 3, Figure 4 and Figure 5, respectively. Upon treatment with standard drugs, surface morphological changes were clearly visible. 

The structural damages were significant as no clear cellular boundaries are visible. Similarly, in the case of newly synthesized compounds, the antimicrobial effect was obvious, as recorded by AFM. The irregular cellular shape, disoriented colonies and lack of clear boundaries signify the antimicrobial efficacies of these compounds.

### 3.2. Cytotoxic Activities

**Figure 6:**
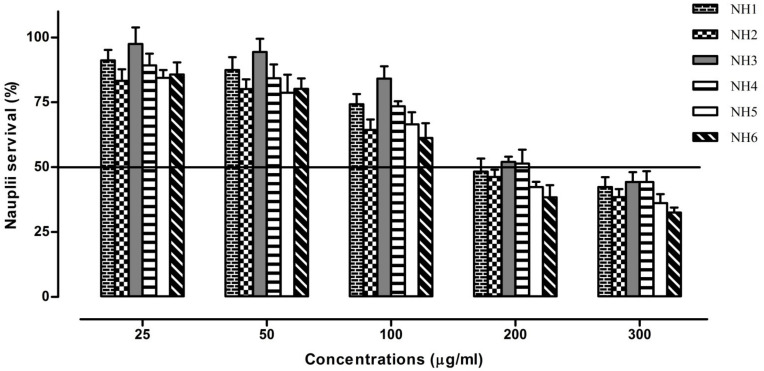
Brine shrimp lethality test for the synthesized hydrazones at various concentrations.

The cytotoxic activities were assessed using brine shrimp lethality assay. The LC_50_ obtained for synthesized compounds are presented in Figure 6. 

The LC_50_ observed for the test compounds were between 162.5 (NH6) and 240 (NH3) µg/mL, indicating their toxic nature as per Meyer’s and Clarkson toxicity scale their LC_50_ is below 1000µg/mL. Since the brine shrimp lethality test has a strong correlation with cytotoxicity in human cancer cells, it can be used for the screening of anticancer potential. However, due to its lower sensitivity to distinguish between strong, moderate and weak effects, more delicate procedures are required for testing on human cancer cell lines [29].

## 4. Discussion

This study reports the synthesis of vanillinaldehyde and salicylaldehyde hydrazones with isoniazid employing azomethine linkage. The detailed principal peaks are given for each compound. The FTIR spectral analysis supported the presence of the characteristic functional groups present in the synthesized compound. The presence of peaks at ῡ = 1577.51–1615.26 cm^−1^ confirmed the formation of an imine linkage (C=N) in all the compounds of the series synthesized from NH1 to NH6. The existence of the ester functional groups (C=O) of NH1 and NH2 was ascertained with the appearance of peaks at ῡ = 1730.65–1733.15 cm^−1^; peaks at ῡ = 3230.42–3294.42 cm^−1^ were assigned to (N-H) in all the compounds of the series. The N-N linkage vibrated at ῡ = 1112.43–1163.08 cm^−1^ and amide linkage (N-C=O) carbonyl group vibrated is ῡ = 1639.49–1745.10 cm^−1^ for all compounds of the series. The presence of an aromatic (C-H) peak appeared at around 2926.01 to 2975.26 cm^−1^. The ^1^H-NMR signals at chemical shifts (*δ*, ppm) of 1.04 to 5.46 are assigned to side alkyl chain in each of these compounds of the series, from NH1 to NH6, which are adjacent to the aromatic ring. The signals at *δ* 3.91, 3.91 and 3.93 ppm are assigned to the aromatic–OCH_3_ protons of NH3, NH5 and NH1, respectively. The resonance peaks between 8.26 ppm and 8.29 ppm in the spectra were assigned to the azomethine (−CH=N-) of these hydrazones. Signals at *δ* 6.97–8.94 ppm are assigned to the aromatic protons. In the 13CNMR spectra of all these hydrazones, four key resonance signals were observed. These are *δ* 143.19-144.02 for azomethine (−CH=N-) and the terminal alkyl groups in all compounds, NH1-NH6, appeared in the range from *δ* 10.49 to 133.11. The carbonyl carbon of the acyl group roughly appeared at *δ* 164.08–168.08 and carbonyl carbon of ester linkage of NH1 and NH2 resonates at *δ* 167.7–168.7, respectively. The signals of −OCH_3_ carbon adjacent to the aromatic ring of NH1, NH3 and NH5 resonates at *δ* 56.62, 56.75 and 57.19, respectively. Peaks of aromatic carbons of both benzene and pyridine rings in all six compounds showing chemical shifts *δ* range from 112 to 152. These spectroscopic data confirmed the successful syntheses of the six hydrazones mentioned above. The *λ*_max_ (nm) values of these six hydrazones were calculated according to their UV-Vis spectra are in the *λ*_max_ range of 320 to 330 nm. 

## 5. Conclusions

The results obtained in this study concluded that the synthesized compounds were of good yields and exhibited appreciable potential due to their antibacterial and cytotoxic properties. Amongst the synthesized compounds, NH3 was found to have promising antibacterial properties, while NH6 was observed to have high cytotoxicity behavior. Studies should be undertaken to assess their anticancer activity using human cancer cell lines to reveal their true potential for therapeutic use. These hydrazones can be further modified for metal coordination for further investigation of other biological properties. 

## Figures and Tables

**Table 1:** Physical data of the synthesized compounds.

S. No.	**Sample Code**	**M.F**	Molecular Structure	**M.P** (°C)	**RF**	%Yield	Color
**1**	NH1	C20H23N3O5	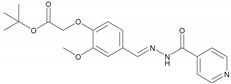	169–170	0.45	68	Light-yellow
**2**	NH2	C19H21N3O4	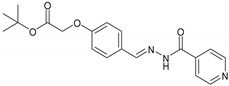	173–174	0.40	69	Light-yellow
**3**	NH3	C19H21N3O3	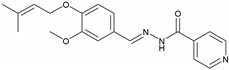	178–179	0.40	70	Light-yellow
**4**	NH4	C18H19N3O2	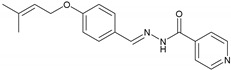	179–180	0.45	66	Light-yellow
**5**	NH5	C_17_H_19_N_3_O_3_	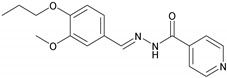	152–153	0.45	71	Light-yellow
**6**	NH6	C_16_H_17_N_3_O_2_	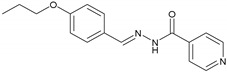	143–145	0.47	63	Light-yellow

**Table 2:** Antibacterial activity via disc diffusion method.

Compound	Antibacterial Activity (Paper Disc Diffusion Method)		
	Zone of Inhibition (mm)		
	*Staphylococcus aureus ATCC 25923* (Gram +ve)	*Bacillus subtilis ATCC 11774* (Gram +ve)	*Escherichia coli ATCC 10536* (Gram −ve)
NH1	20	14	25
NH2	15	14	14
NH3	24	22	33
NH4	20	23	21
NH5	22	25	24
NH6	17	23	22
Amikacin	24	30	38

**Table 3:** Minimum inhibitory concentration of synthesized compounds against *Staphylococcus aureus*.

Conc. µg/mL	% Inhibition of S. *aureus* ATCC 25923						
	Ceftriaxone	NH1	NH2	NH3	NH4	NH5	NH6
**25**	30 ± 0.8	-	11 ± 1.0	24 ± 0.7	16 ± 0.5	5 ± 0.4	-
**50**	55 ± 0.3	10 ± 0.6	21 ± 1.2	35 ± 1.2	24 ± 1.0	12 ± 0.4	5.5 ± 0.5
**100**	80 ± 1.2	25 ± 0.7	42 ± 2	44 ± 2.0	38 ± 1.5	35 ± 0.7	25 ± 0.5
**200**	96 ± 1.0	55 ± 1	50 ± 2	60 ± 1.5	55 ± 0.9	58 ± 1.1	52 ± 1.6
**300**	-	63 ± 1	63 ± 1.5	78 ± 1	71 ± 2.0	77 ± 1.5	72 ± 1.2
**400**	-	69 ± 2	74 ± 2	89 ± 1.5	89 ± 1.5	-	80 ± 1.0
**500**	-	80 ± 1.5	-	-	-	-	-

**Table 4:** Minimum inhibitory concentration of synthesized compounds against *Bacillus subtilis*.

Conc. µg/mL	% Inhibition of B. *subtilis* ATCC 11774						
	Ceftriaxone	NH1	NH2	NH3	NH4	NH5	NH6
**25**	40 ± 0.5	-	4 ± 0.2	12 ± 0.4	10 ± 0.7	-	-
**50**	55 ± 0.7	5 ± 0.3	12 ± 0.3	20 ± 0.7	18 ± 0.6	10 ± 0.5	10 ± 0.6
**100**	79 ± 1.2	26 ± 0.3	22 ± 0.3	28 ± 0.8	29 ± 0.8	26 ± 0.5	23 ± 0.7
**200**	92 ± 1.2	44 ± 0.4	44 ± 0.5	53 ± 0.7	50 ± 1.1	53 ± 0.6	52 ± 0.8
**300**	-	60 ± 0.5	60 ± 0.5	74 ± 0.9	68 ± 1.0	73 ± 0.8	70 ± 1.1
**400**	-	75 ± 0.5	81 ± 0.6	84 ± 0.9	77 ± 0.8	-	-

**Table 5:** Minimum inhibitory concentration of synthesized compounds against *Escherichia Coli*.

Conc. µg/mL	% Inhibition of E. *coli* ATCC 10536						
	Amoxicillin	NH1	NH2	NH3	NH4	NH5	NH6
**25**	40 ± 0.5	9 ± 0.5	-	-	-	10 ± 0.6	5 ± 0.2
**50**	66 ± 0.5	18 ± 0.5	-	36 ± 0.5	5 ± 0.5	18 ± 0.6	14 ± 0.5
**100**	85 ± 0.6	40 ± 0.4	10 ± 0.3	50 ± 0.5	25 ± 0.5	34 ± 0.3	28 ± 0.5
**200**	96 ± 1.0	60 ± 0.5	25 ± 0.3	65 ± 0.2	54 ± 0.4	58 ± 0.6	50 ± 0.7
**300**	-	79 ± 1.0	55 ± 0.5	85 ± 1.2	75 ± 0.8	79 ± 0.8	73 ± 0.7
**400**	-	-	70 ± 0.3	-	-	-	-

## Data Availability

All the available data are incorporated in this manuscript.
